# An analysis *in vivo* of intracanal bacterial load before and after chemo-mechanical preparation: A comparative analysis of two irrigants and two activation techniques

**DOI:** 10.4317/jced.52585

**Published:** 2016-02-01

**Authors:** Cristina Rico-Romano, Álvaro Zubizarreta-Macho, María-Rosario Baquero-Artigao, Jesús Mena-Álvarez

**Affiliations:** 1 Associate Professor. Master’s Degree in Clinical Endodontics and Periapical Microsurgery. Faculty of Health Sciences. Alfonso X el Sabio University. Madrid, Spain; 2 Vice Dean. Faculty of Health Sciences. Alfonso X el Sabio University. Madrid, Spain; 3 Head Academic Master Degree in Endodontics. Faculty of Health Sciences. Alfonso X el Sabio University. Madrid, Spain

## Abstract

**Background:**

The goals of this randomized double-blind trial were to assess the antimicrobial activity *in vivo* of Sodium hypochlorite (NaOCl) vs. chlorhexidine gluconate (CHX) used in combination either with EndoActivator® or IRRI S® files in patients with apical periodontitis.

**Material and Methods:**

A total of 120 patients with apical periodontitis (in single or multiple root canals) were randomly assigned to the four irrigation protocols outlined below: Group A: 5.25% sodium hypochlorite (NaOCl) + EndoActivator®; Group B: 5.25% NaOCl + IRRI S® files; Group C: 2% chlorhexidine gluconate (CHX) + EndoActivator®; Group D: 2% CHX + IRRI S® files. Paper points were used to collect microbiological samples before (1A samples) and after (1B samples) irrigation. Viable colony-forming units (CFU) were quantified twice: (1) without speciation, and (2) only for *Enterococcus Faecalis*(*EF*). Statistical analysis was performed using SPSS 22.0 for Windows.

**Results:**

No significant differences were observed between NaOCl and CHX in the reduction of CFU; in fact, reduction was < 93% for the two irrigants. Conversely, statistically significant differences were found between the two activation techniques (sonic and ultrasonic) in the reduction of *Enterococcus Faecalis*(*EF*). Thus, the effectiveness of ultrasonic activation was significantly higher (< 93%; *p*=0.012) as compared to sonic activation. Following the combination of the two irrigants with the two activation techniques (groups A, B, C and D), significant differences were observed between group A and B (*p*=0.025) in the reduction of EF populations, reaching up to 94%.

**Conclusions:**

NaClO and CHX are effective in reducing intracanal bacterial load. Ultrasonic activation is the most effective activation technique in reducing *EF* populations.

** Key words:**Chlorhexidine gluconate, sodium hypochlorite, ultrasonic irrigation, sonic irrigation, apical periodontitis, Enterococcus faecalis.

## Introduction

Apical periodontitis is the defense mechanism the human body has developed to keep destruction of the dental pulp and microbial infection of the root canal system from spreading beyond the apical foramen and allow periapical tissue repair (1). The treatment of choice for periodontitis involves the chemo-mechanical preparation of root canals to remove or reduce the microbial load until it is compatible with periapical health. However, this clinical procedure has been proven inefficient, since 40-60% of residual bacteria persist after root canal treatment (2).

The intricate nature of the apical third of the root canal system hinders root canal treatment (3). Consequently, a number of irrigant activation techniques have been developed to remove the smear layer loosened by mechanical instrumentation and improve the antimicrobial effect of irrigating solutions (3). In the last decades, irrigants have been extensively studied and a variety of irrigating solutions have been proposed for root canal treatment. However, since sodium hypochlorite (NaClO) was introduced in endodontics by Walker in 1936, it is still the irrigant of choice due to its potent antimicrobial action and its ability to dissolve necrotic tissue (4). Notwithstanding its effectiveness, a range of adverse effects –such as inflammation, haematomas and ultimately necrosis and paresthesia– have been associated with irrigation of periapical tissue with NaClO (5). Also, Rasimick *et al.* observed that NaClO does not eradicate microbia from root canals completely (6). This fact evidences the need for the development of new irrigation techniques that overcome the limitations of NaClO and keep its properties. Chlorhexidine gluconate (CHX) arises as a plausible option due to its potent antimicrobial activity. A range of studies have been conducted to compare the effectiveness of CHX vs. NaClO, with conflicting results (7). On the one hand Vianna *et al.* demonstrated that the antimicrobial effect of NaClO is superior to that of CHX (8). On the other hand, Ferraz *et al.* reported that CHX has a more potent antimicrobial activity than NaClO (9). However, CHX has an important property: substantivity. In 2002, Basrani *et al.* concluded that canal dressing with 2% CHX for one week may provide residual antimicrobial activity (10).

Such inconsistency of results evidences the need for a study that compares the clinical effectiveness of CHX vs. NaClO and sonic vs. ultrasonic irrigation to determine the most effective combination for the treatment of the root canal system.

## Material and Methods

-Study design

A randomized, double-blind trial was performed involving a sample of 120 patients (sample size was estimated basing on the results obtained by Vianna *et al.*) (8). This trial was conducted in accordance with the ethical principles of the Declaration of Helsinki and following Best Practice guidelines. The study was performed at the Clínica Universitaria Odontológica Alfonso X el Sabio, Madrid, Spain between July 2011 and January 2013. The informed consent form was approved by the Ethics Committee of the Alfonso X el Sabio University (01/2011). Randomization of the patients into four groups was performed using Epidat version 3.1, OPS-OMS, A Coruña, Spain: Group A: irrigation with 5.25% NaClO with sonic activation for 30 seconds at 10,000 revolutions per minute (rpm) (EndoActivator®, Dentsply Maillefer®, Ballaigues, Switzerland). Group B: irrigation with 5.25% NaClO with ultrasonic irrigation for one minute. (IRRI S®, VDW® GmbH, Munich, Germany). Group C: irrigation with 2% CHX with sonic irrigation. Group D: irrigation with 2% CHX with ultrasonic irrigation. All solutions were prepared in the El Globo phar-macy, Madrid. Inclusion and exclusion criteria are shown in  Table 1.

**Table 1:**
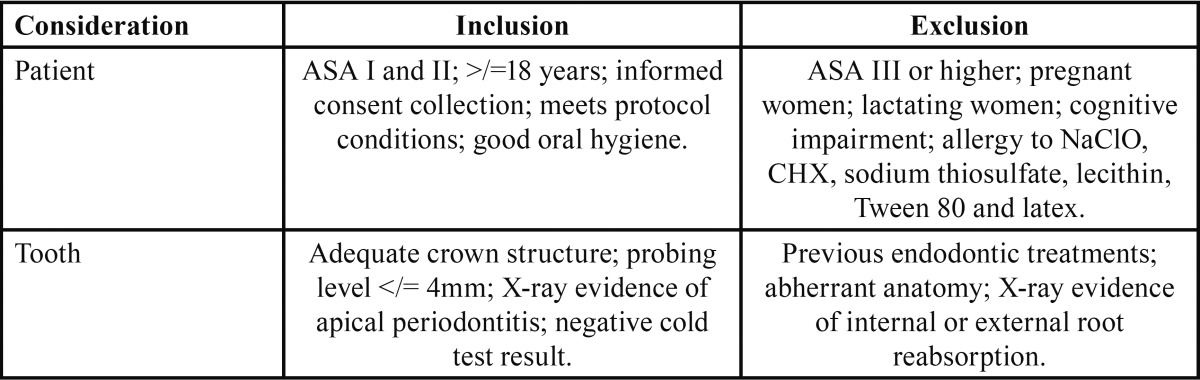
Inclusion and Exclusion Criteria Considered for Enrollment of Subjects in the Study.

-Clinical procedure

After infiltration anesthesia, root canals were treated with total isolation using Hygenic® dental dam, Coltene® Whaldent Gruppe, Altstätten, Switzerland). The involved crown and the dental dam were irrigated with 30% H2O2 for 30 seconds and with 2.5% NaClO for another 30 seconds. Deactivation was achieved by final irrigation with 5% sodium thiosulfate. Subsequently, the pulp chamber was opened to access the root canal system. Then, a baseline sample was collected (Sample 1A) by inserting sterilized 15-mm paper points (Dentsply Maillefer, Ballaigues, Switzerland) into the root canal system. Next, the sample was transferred into a sterile Eppendorf pellet with 1ml saline.

Canal instrumentation was performed with K-Flexofile® files to a size 20/.02 file (Dentsply Maillefer, Ballaigues, Switzerland). Root canal working length was measured using the electronic appex locator Root ZX® (Morita®, Tokio, Japan) and verified with a working length radiograph. Root canals were shaped using the Protaper Universal®root instrumentation system (Dentsply Maillefer, Ballaigues, Switzerland) to a size 25/.06 file. During instrumentation, disinfection was performed with the corresponding irrigant and irrigation technique. Then, irrigants were deactivated: 5% sodium thiosulfate for NaClO and lecithin and Tween 80 for CHX. Following root canal obturation, a final sample (Sample 1B) was collected using sterilized 15-mm paper points. Finally, the chamber opening was provisionally obturated with cavity cement (Cavit™, 3M ESPE, Saint Paul, MN, USA).

-Microbiological procedure

The two microbiological samples collected from each patient were submitted to the microbiology laboratory of Alfonso X University for analysis. Samples 1A and 1B were diluted at a concentration of 1/10 to facilitate microbiological counting.

The diluted 1A and 1B samples were placed on Agar Sangle plates (placas de Columbia Agar with 5% sheep blood, 770418 Dis-malab SL) and incubated aerobically in a stove at 37ºC for 48 hours for colony-forming units (CFU) counting.

The undiluted 1A and 1B samples were plated on bile esculin agar (enterococcosel agar, 770812 Dismalab SL) for the detection of *Enterococcus faecalis*. Next, the plates were incubated at 37ºC for 48 hours for colony-forming units counting of *Enterococcus faecalis*.

-Statistical analysis

All variables of interest were recorded for statistical analysis with SPSS 22.00 for Windows. Descriptive statistics are expressed as means and standard deviation for quantitative variables and as absolute numbers and percentages for qualitative variables. Comparative analysis was performed by comparing the mean colony-forming unit count for each group before and after the intervention using Mann-Whitney U test, since variables did not have a normal distribution. A *p* </= 0.05 was considered statistically significant.

## Results

The bacterial population decreased significantly in the four study groups ( Table 2). No statistically significant differences were found between NaClO and CHX regarding CFU reduction without speciation (*p*=0.853) or in the reduction of *Enterococcus faecalis* populations (*p*=0.777). In both cases reductions were above 93%.

**Table 2:**
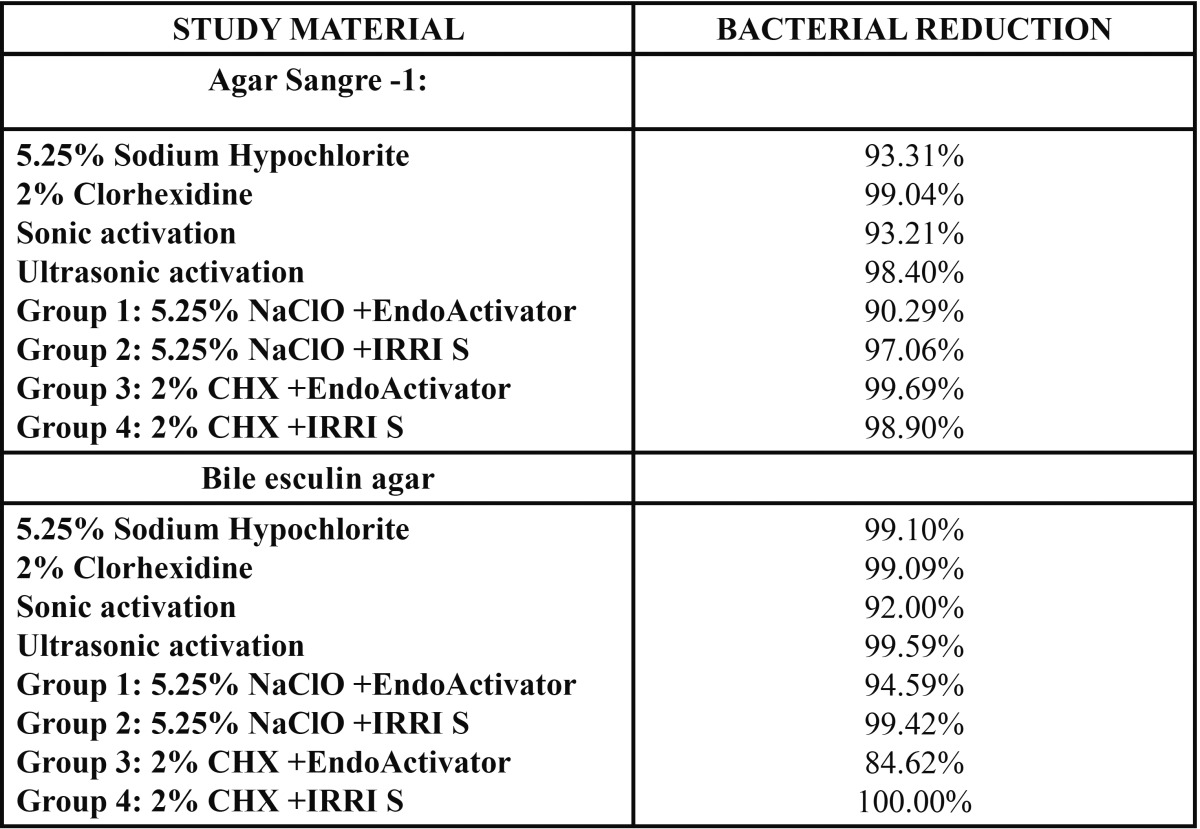
Percentage of bacterial reduction by study system.

No statistically significant differences were either found between sonic and ultrasonic activation regarding CFU reduction without speciation (*p*=0.112). However, the ultrasound groups (groups B and D) showed a statistically significant reduction in *Enterococcus faecalis* populations (*p*=0.012). Nevertheless, reductions were above 92% in all groups.

As to irrigant/activation technique combinations, groups A and B showed statistically significant decreases in *Enterococcus faecalis* populations (*p*=0.025).

## Discussion

The causative role of bacteria in the pathogenesis of apical periodontitis underlines the goal of elimination of bacteria as a critical step in root canal therapy (11,12). Bacterial elimination is attempted by mechanical instrumentation, irrigation with antibacterial agents, and medication with intracanal dressing (12).

A number of authors have analyzed the efficacy of the different antimicrobial agents proposed in the literature. On the one hand, several authors have demonstrated the effectiveness of NaClO and CHX *in vivo* (7,13) and *ex vivo* (14-16) in reducing CFU count, with no significant differences between these two irrigants. On the other hand, studies performed *in vitro* have evidenced statistically significant differences between NaClO and CHX. These results may be due to the use of pure bacterial cultures and unfavourable environmental conditions for the growth of resistant biofilms (9,17).

Some factors such as temperature, dilution concentration and time of exposure have an impact on the properties of irrigants (18). However, since to take advantage of external factors the penetation of irrigants must be boosted, we also assessed the effectiveness of different irrigant activation techniques. Some authors have demonstrated the ability of ultrasounds to facilitate the penetration of irrigants (19,20). Thus, these authors have concluded that ultrasound activation is the most effective technique to remove microorganisms and debris from the root canal system (21-24).

Conversely, a number of authors have reported that sound activation is the most effective technique for the disinfection of the root canal system, provided that the irrigant is delivered at the adequate concentration and for the appropriate time of exposure (3,4,25,26). Other authors such as Huffaker *et al.* did not find any significant differences between conventional irrigation and sonic activation (1).

Finally, there are studies of the efficacy of sonic activation vs. ultrasonic activation where no significant differences were found (27,28). These results are consistent with those obtained in our study (27,28).

## Conclusions

The following conclusions can be drawn from the results obtained in this study:

1. For a successful removal of bacteria from root canals, instrumentation should be performed concomitantly with a disinfecting agent activated by vibration.

2. As to the type of vibration, ultrasound activation has proven to be the most effective method for the eradication of EF (*p* = 0.012).

3. When comparing the four study groups, statistically significant differences were found in UFC reduction between Group A (NaClO + sonic activation) and Group B (NaClO + ultrasounds) (*p* < 0.025), being the latter more effective. However, no significant differences were found between Group B and groups C and D (CHX).
